# Analysis of the Expression and Prognostic Value of Annexin Family Proteins in Bladder Cancer

**DOI:** 10.3389/fgene.2021.731625

**Published:** 2021-08-13

**Authors:** WenBo Wu, GaoZhen Jia, Lei Chen, HaiTao Liu, ShuJie Xia

**Affiliations:** Department of Urology, Shanghai General Hospital (Shanghai First People’s Hospital), Shanghai Jiao Tong University School of Medicine, Shanghai, China

**Keywords:** annexin, NMIBC, MIBC, prognosis, public databases

## Abstract

**Background:**

Bladder cancer (BC) is the most common tumor of the urinary system. Non-muscle-invasive bladder cancer (NMIBC) has a high recurrence rate after surgery, and patients with muscle-invasive bladder cancer (MIBC) have poor quality of life after radical surgery. Understanding the molecular mechanism of bladder cancer is helpful for providing a more appropriate treatment approach. Annexins are calcium-binding proteins and play an important role in different tumor cells. However, the role of the annexin family in bladder cancer has not been studied in detail.

**Methods:**

ONCOMINE, UALCAN, TIMER2.0, Kaplan-Meier Plotter, cBioPortal, and WebGestalt were utilized in this study.

**Results:**

ANXA2, ANXA3, ANXA4, ANXA8, and ANXA9 were significantly increased in bladder tumor tissues, while ANXA6, ANXA7, and ANXA11 were significantly decreased. ANXA1, ANXA2, ANXA3, ANXA5, ANXA6, ANXA7, and ANXA9 had prognostic value in bladder cancer. In addition, specific annexins were specifically expressed in different subtypes of MIBC and were related to the histological morphology of bladder tumors. ANXA1, ANXA2, ANXA3, ANXA5, ANXA6, ANXA7, and ANXA8 were highly expressed in basal-subtype MIBC, while ANXA4, ANXA9, ANXA10, and ANXA11 were mainly expressed in luminal-subtype MIBC. Finally, we analyzed the possible mechanisms of ANXAs in different subtypes of bladder cancer through GO and KEGG analyses and the correlation between ANXAs and immune infiltration in the tumor microenvironment.

**Conclusion:**

Taken together, our results indicate that annexins might play important roles in BC and have the potential to be used as markers for subtype classification.

## Introduction

Bladder cancer (BC) is the most common genetic disease of the urinary system, and studies have shown that the progression of disease is related to the accumulation of multiple genetic and epigenetic mutations (Kim et al., 2010). The genes TP53, Rb1, and FGFR3 have been verified to be closely related to the prognosis of bladder cancer (Grossman et al., 1998; van Rhijn et al., 2001). According to the American Joint Committee on Cancer TNM Oncology Staging System, bladder cancer can be divided into non-muscle-invasive bladder cancer (NMIBC pTa-pT1) and muscle-invasive bladder cancer (MIBC ≥ pT2). MIBC presents with rapid progression, metastasis, and poor prognosis, and approximately 30% of newly diagnosed bladder cancers present muscle-infiltration (Chou et al., 2016). The treatment of NMIBC and MIBC is different. Transurethral resection of the bladder tumor (TurBT) combined with intravesical instillation of chemotherapy drugs is the main treatment for NMIBC. However, for MIBC patients, radical cystectomy and systemic chemotherapy, which have a serious impact on quality of life, are required. In addition, 50% of patients may develop tumor progression and distant metastasis (van Lingen et al., 2013; Chou et al., 2016). In recent years, immunotherapies, such as immune checkpoint inhibitors, have shown long-term durable responses and tolerable safety profiles in several clinical trials. However, approximately 70–80% of patients may be unresponsive to immune checkpoint inhibition (Kim and Seo, 2018). Therefore, understanding the molecular mechanism of bladder cancer and identifying effective biomarkers and potential therapeutic targets are of great significance for the treatment and diagnosis of BC.

Annexins are a superfamily of secreted proteins that exist in the cytoplasm and attach to the phospholipid membranes of cells and are currently classified into five classes (groups A–E). Group A is present in vertebrates, and 13 group A members (annexin A1–A13) are present in human organs (Iglesias et al., 2002). ANXA1 was the first to be studied, and studies have shown that ANXA1 is involved in the regulation of inflammation and can affect T cell proliferation (Han et al., 2020). In addition, ANXA1 is highly expressed in different cancers, such as hepatocellular carcinoma, lung cancer, and colorectal cancer (Lecona et al., 2008; Biaoxue et al., 2012; Suo et al., 2012). In contrast, its expression is decreased in cervical cancer and prostate cancer (Patton et al., 2005; Moghanibashi et al., 2012). Studies in urinary system cancer have shown that renal cancer patients with low expression of ANXA1 have a better prognosis, and Yu’s study showed that high expression of ANXA1 was associated with recurrence of bladder cancer (Yu et al., 2014; Yamanoi et al., 2019). In addition, ANXA2 is also a protein that has been studied extensively, and studies have shown that ANXA2 is highly expressed in urothelial carcinoma and is associated with prognosis (Zhang et al., 2014; Wang et al., 2015). We used TCGA data to find that in addition to ANXA1 and ANXA2, other ANXA family proteins (ANXA3, ANXA5, ANXA6, and ANXA9, which have not been studied) are also closely related to the prognosis of bladder cancer. Therefore, we analyzed the mRNA and protein expression of different annexins in bladder cancer and their correlation with the histological morphology and clinical stage of bladder cancer. Furthermore, we performed enrichment analysis of 25 genes related to each ANXA protein to clarify its potential functions. Since annexins regulate inflammation, we also analyzed the relationship between the expression of ANXA family members and immune cell infiltration. This study shows that ANXA family proteins are closely related to the development of the bladder cancer and have high prognostic value that will be beneficial to the diagnosis and treatment of bladder cancer.

## Materials and Methods

### mRNA Expression Level of ANXA Family Proteins

UALCAN^1^ and Oncomine^2^ were used to analyze the expression of ANXA mRNA. The mRNA expression in tumor tissues and normal tissues was analyzed by UALCAN, and the mRNA expression difference between MIBC and NMIBC was verified with three datasets from Oncomine.

### Protein Expression Level of ANXA Family Proteins

Protein expression levels in different tumors can be presented intuitively with immunohistochemistry data through the HPA platform^3^. In this study, we compared the expression levels of ANXA family proteins in normal tissues and in tissues from bladder cancers of different grades. Protein expression were quantified using a visual grading system based on the extent of staining (percentage of positive tumor cells graded on scale from 0 to 3: 0, none; 1, <25%; 2, 25–75%; 3, >75%) and the intensity of staining (graded on a scale of 0–3: 0, none; 1, weak staining; 2, moderate staining; and 3, strong staining). The combination of extent (E) and intensity (I) of staining was obtained by the product of EI (Maréchal et al., 2009). Extent and intensity information of staining could be obtained on the HPA. Three sections of each type of sample (normal, low grade BC, and high grade BC) were selected randomly and the mean value was used for statistical analysis.

### Correlation Between the Expression Levels of ANXA Family Proteins and Clinicopathological Features

In this study, we used UALCAN to analyze the correlation between ANXA mRNA expression and clinical parameters of bladder cancer patients, including individual tumor stage and molecular subtype. The significance of the differences is marked in the figure.

### Survival Analysis

Kaplan-Meier Plotter^4^ provides survival data from patients with 21 different cancer types. In this article, we evaluated the relationship between different ANXA mRNA expression levels and overall survival time. According to the mRNA expression level, patients were divided into high expression and low expression groups based on the appropriate cutoff value determined by the Kaplan-Meier plotter platform. The results reflect the prognostic utility of different ANXA proteins. A *P*-value less than 0.05 was considered to indicate a statistical difference.

### Correlation Analysis of ANXA Expression and Characteristic Subtype Gene Expression in Bladder Cancer

Timer 2.0^5^ was used to analyze the correlation between the expression of membrane actin and the expression of luminal subtype characteristic genes (FOXA1, GATA3, and KRT20) as well as basal subtype-related genes (KRT5, KRT6, and KRT14).

### GO and KEGG Analyses of ANXA-Related Genes

We analyzed 25 positively co-expressed genes of each ANXA protein using cBioPortal^6^. According to different groups, online GO and KEGG analyses were performed using WebGestalt^7^.

### Correlation Analysis of ANXA Expression and Immune Cell Infiltration

Timer 2.0 also provides a platform to perform a correlation analysis between the expression of a single gene and the infiltration of immune cells. Therefore, we analyzed the relationship between the expression of each ANXA protein and the infiltration of six major immune cells in bladder cancer.

### Statistical Analysis

Data are shown as means ± standard deviation (SD). All statistical analyses were carried out using SPSS (version 23, IBM; Armonk, NY, United States). Differences between groups were analyzed using the two-tailed Student’s *t*-test, one-way ANOVA. Statistical significance was set at *P* < 0.05.

## Results

### mRNA Expression of ANXA Family Members in Patients With BC

**FIGURE 1:**
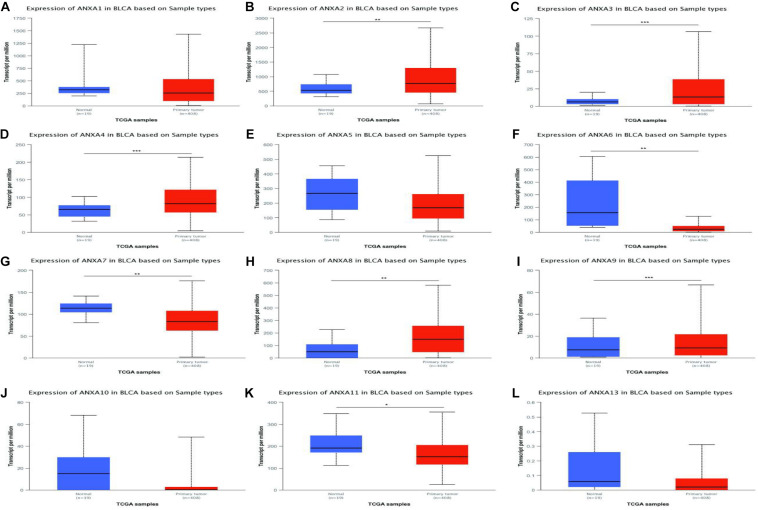
mRNA expressions of different ANXA family members in bladder cancer (TCGA database) **(A–L)**. (*p < 0.05; **p < 0.01; ***p < 0.001).

We used the TCGA database to analyze the mRNA expression of ANXA1–11 and ANXA13 in bladder cancer tissues and normal tissues. As shown in Figure 1, the expression levels of ANXA2, ANXA3, ANXA4, ANXA8, and ANXA9 were significantly increased in tumor tissues compared with normal tissues. Conversely, the expression levels of ANXA6, ANXA7, and ANXA11 were significantly reduced in tumor tissues. In addition, ANXA1, ANXA5, ANXA10, and ANXA13 showed no significant difference between the two groups. We also compared the expression of different ANXA mRNAs between NMIBC and MIBC (Supplementary Table 1). As seen in Supplementary Table 1, we selected three datasets from the Oncomine database from the studies of Sanchez-Carbayo, Dyrskjot, and Lee, and the datasets included 157, 60, and 256 bladder cancer samples, respectively (Accession numbers of dataset and reasons for selection were provided in Supplementary Table 3). The Sanchez-Carbayo dataset showed that the mRNA expression levels of ANXA1, ANXA3, ANXA5, ANXA6, ANXA7, and ANXA8 in MIBC were significantly higher than those in NMIBC, while the mRNA expression levels of ANXA9, ANXA10, ANXA11, and ANXA13 were significantly lower in MIBC than in NMIBC. In Dyrskjot’s dataset, the expression levels of ANXA1, ANXA2, ANXA3, ANXA5, and ANXA6 were the same in MIBC, while the expression levels of ANXA13 were decreased, and the other expression levels were not significantly changed. Finally, in Lee’s dataset, the expression of ANXA1, ANXA2, ANXA3, ANXA5, and ANXA8 was significantly increased in MIBC patients, while the expression of ANXA10 and ANXA11 was decreased in MIBC patients. It should be noted that there were no expression data for ANXA9 in Lee’s dataset.

### Protein Expression of Different ANXA Family Members in Bladder Cancer

**FIGURE 2:**
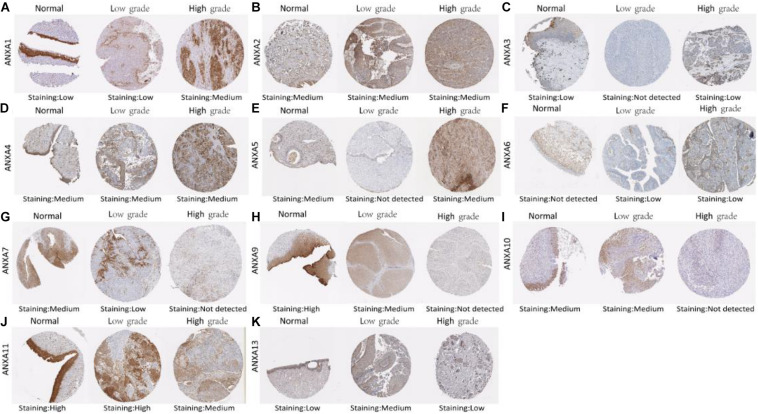
Representative immunohistochemistry images of different ANXA family members in normal bladder tissues, low grade BC tissues and high grade BC tissues (HPS database) **(A–K)**.

Immunohistochemistry can reflect the expression of proteins in tissues. We used HPA to investigate differences in the expression of ANXA protein members between normal tissues and low-grade and high-grade bladder cancers. The staining degree could be divided into low medium and high. The results of staining degree were provided by HPA, which was based on the results of staining intensity and quantity. Representative sections were shown in Figure 2 to show the expression trend of ANXA proteins. As it shown, ANXA1 was more strongly stained in high-grade bladder cancer than in low-grade bladder cancer. Similar expression differences were observed in ANXA3 and ANXA5. The expression of ANXA7 was decreased in tumor tissues compared with normal tissues and was not detected in high-grade bladder cancer. Similarly, the expression of ANXA9–11 and ANXA13 in high-grade bladder cancer was lower than that in low-grade bladder cancer. The expression data of ANXA8 were not found in HPA. The quantitative analysis results were shown in Supplementary Tables 4, 5, results indicated that protein expression level of ANXA9 and ANXA11 was significantly decreased in bladder tumor tissues compared with normal tissues (*P* = 0.049; *P* = 0.0014), and the expression level of ANXA1 and ANXA3 in high grade BC were significantly increased than those in low grade bladder cancer (*P* = 0.0075; *P* = 0.008).

### Prognostic Value of ANXA Protein mRNA Expression

**FIGURE 3:**
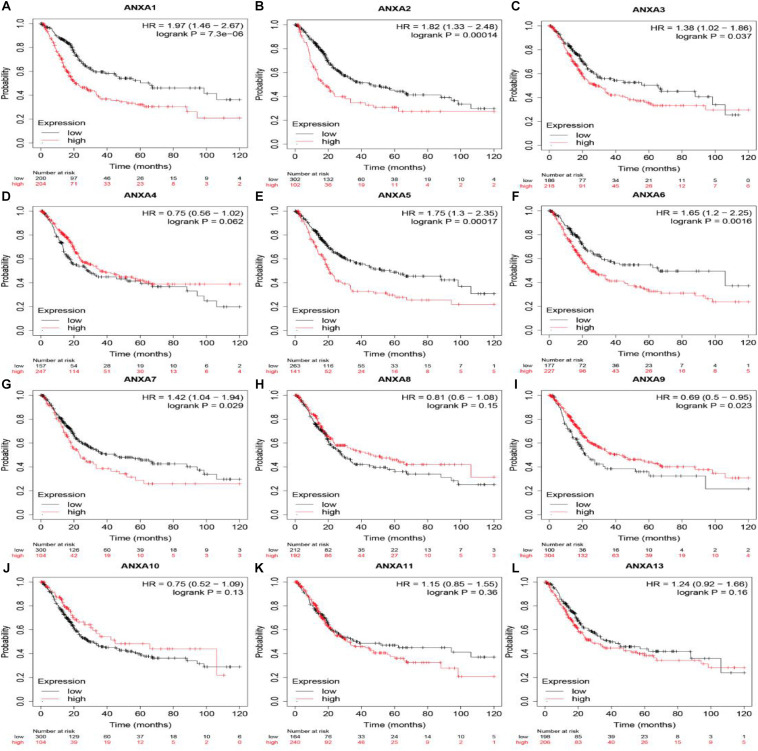
OS of mRNA expression of ANXA family members in BC patients. Survival curves comparing the high and low expression of ANXA family members in BC patients in Kaplan-Meier Plotter **(A–L)**.

The Kaplan-Meier Plotter platform used TCGA data to analyze the prognosis of different genes. As shown in Figure 3, BC patients with higher mRNA expression of ANXA1 (HR = 1.97, logrank *P* = 7.3e-06), ANXA2 (HR = 1.82, logrank *P* = 1.4e-04), ANXA3 (HR = 1.38, logrank *P* = 0.037), ANXA5 (HR = 1.75, logrank *P* = 1.7e-04), ANXA6 (HR = 1.65, logrank *P* = 1.6e-03), and ANXA7 (HR = 1.42, logrank *P* = 0.029) had shorter OS. Conversely, patients with higher expression of ANXA9 (HR = 0.69, logrank *P* = 0.023) had a longer overall survival. In addition, there was no significant correlation between the mRNA expression level of ANXA4 (HR = 0.75, logrank *P* = 0.062), ANXA8 (HR = 0.81, logrank *P* = 0.15), ANXA10 (HR = 0.75, logrank *P* = 0.13), ANXA11 (HR = 1.15, logrank *P* = 0.36), or ANXA4 (HR = 1.24, logrank *P* = 0.16) and overall survival.

### Correlation Between the mRNA Expression of Different ANXA Members and the Bladder Cancer Stage of Patients

**FIGURE 4:**
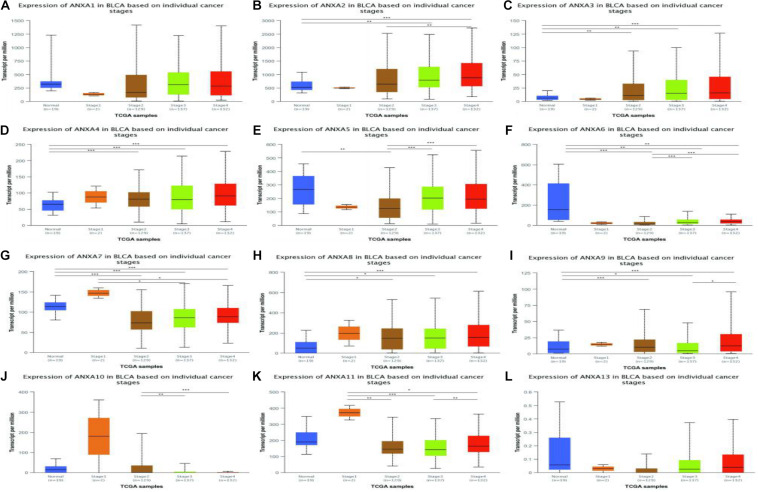
Correlation between the mRNA expression of different ANXA members and the bladder cancer stage of patients in UALCAN database. The mRNA expression of ANXA family members in normal individuals or in BC patients in stages 1, 2, 3 or 4 **(A–L)**. (*p < 0.05; **p < 0.01; ***p < 0.001).

Next, we again used the UALCAN database to explore the relationship between ANXA protein expression and bladder cancer tumor stage. As shown in the Figure 4, the expression levels of ANXA2, ANXA5, and ANXA6 were positively correlated with stage, and the mRNA expression level increased with increasing stage. However, the expression levels of ANXA7, ANXA10, and ANXA11 were higher in early-stage patients. The expression levels of ANXA10 in stage T2 patients were significantly higher than those in stage T3–4 patients, while ANXA7 and ANXA11 were mainly expressed in stage T1 patients.

### Expression of ANXA Members in Different Subtypes of Bladder Cancer

**FIGURE 5:**
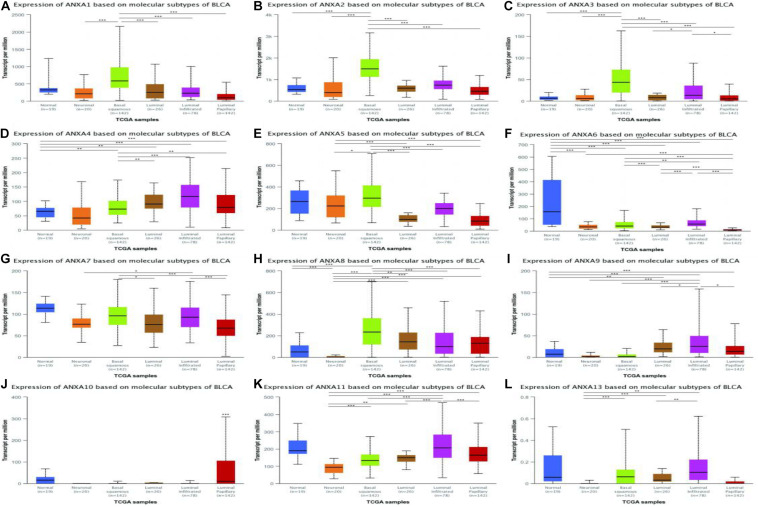
Correlation between the mRNA expression of different ANXA members and molecular subtypes of MIBC patients in UALCAN database. The mRNA expression of ANXA family members in normal individuals or in MIBC patients in five subtypes **(A–L)**. (*p < 0.05; **p < 0.01; ***p < 0.001).

**FIGURE 6:**
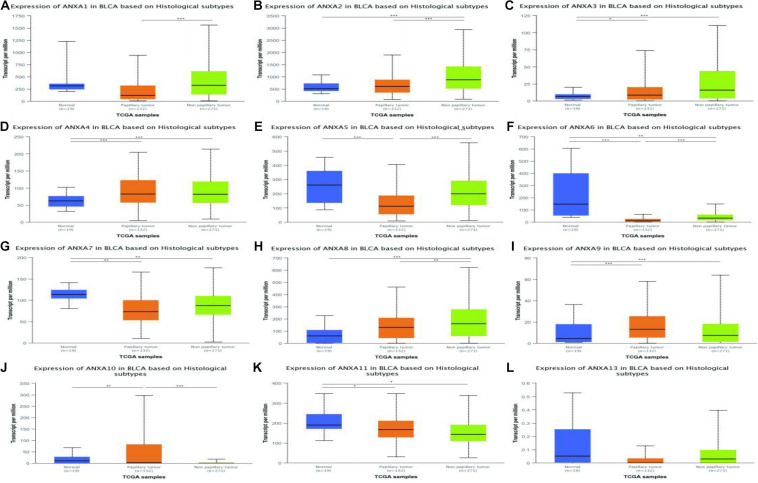
Correlation between the mRNA expression of different ANXA members and histological subtypes of MIBC patients in UALCAN database. The mRNA expression of ANXA family members in normal individuals or in MIBC patients in two histological subtypes **(A–L)**. (*p < 0.05; **p < 0.01; ***p < 0.001).

In a TCGA study from 2017, MIBC bladder cancer was divided into five subtypes: luminal-papillary, luminal, luminal-infiltrated, basal-squamous, and neuronal (Robertson et al., 2017). In the UALCAN platform, samples are from the TCGA database and can be classified based on molecular subtypes. As shown in Figure 5, the expression of ANXA1–3 was significantly higher in basal-squamous-subtype bladder cancer than in other subtypes and had obvious specificity. ANXA5 was mainly expressed in basal-squamous and neuronal types of bladder cancer. Compared with that in normal tissues, the expression of ANXA6 was significantly reduced in five subtypes of MIBC, without obvious subtype specificity. In Figure 5H, the expression of ANXA8 in neuronal subtypes was significantly reduced. However, ANXA9–11 and ANXA13 were mainly expressed in luminal subtypes, with ANXA10 having the highest and most specific expression in luminal-papillary types. In addition, the mRNA expression of ANXA members in histological subtypes were shown in Figure 6, and the results show that ANXA1, ANXA2, ANXA5, ANXA6, and ANXA8 were significantly expressed in non-papillary bladder cancer, while ANXA9, ANXA10, and ANXA11 were mainly expressed in papillary bladder cancer, of which ANXA10 was significant.

### Correlation Between ANXA and Subtype Gene Expression

**FIGURE 7:**
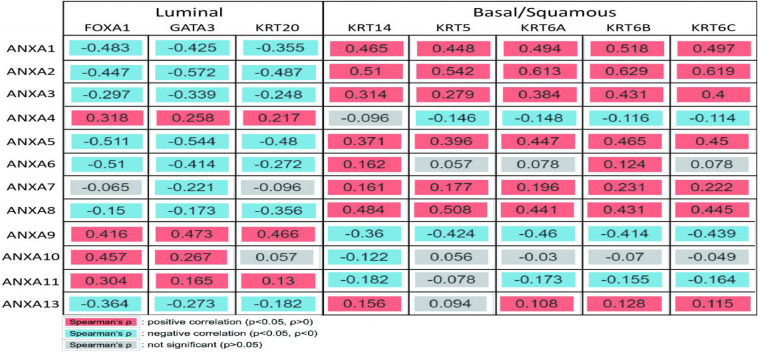
Correlation between ANXA and subtype gene expression. ANXA1, ANXA2, ANXA3, ANXA5, ANXA6, ANXA8, and ANXA13 were significantly positively correlated with the expression of basal type-specific genes and negatively correlated with the expression of luminal type-specific genes. The expression of ANXA4, ANXA9, ANXA10, and ANXA11 showed the opposite patterns.

To further analyze the expression of ANXA family proteins in different MIBC molecular subtypes, we studied the correlation between the expression of ANXA proteins and specific genes of different subtypes. According to TCGA classification and A Kamoun et al.’s study, urothelial differentiation markers such as FOXA1, GATA3, and KRT20 are highly expressed in luminal-subtype bladder cancer, while FOXA1 and GATA3 are not expressed but KRT5, KRT6, and KRT14 are expressed in basal-subtype bladder cancer. As shown in the Figure 7, the expression levels of ANXA1, ANXA2, ANXA3, ANXA5, ANXA6, ANXA8, and ANXA13 were significantly positively correlated with the expression of basal type-specific genes and negatively correlated with the expression of luminal type-specific genes. However, the expression of ANXA4, ANXA9, ANXA10, and ANXA11 showed the opposite patterns. We found that ANXA1, ANXA2, ANXA3, ANXA5, ANXA6, ANXA8, ANXA13, ANXA4, ANXA9, ANXA10, and ANXA11 may have obvious heterogeneity in expression in bladder tumors. The expression of ANXA1, ANXA2, ANXA3, ANXA5, ANXA6, ANXA8, and ANXA13 was closely related to basal-subtype bladder cancer, while the expression of ANXA4, ANXA9, ANXA10, and ANXA11 was closely related to luminal-subtype BC.

### GO and KEGG Enrichment Analyses of ANXA Family Members

**FIGURE 8:**
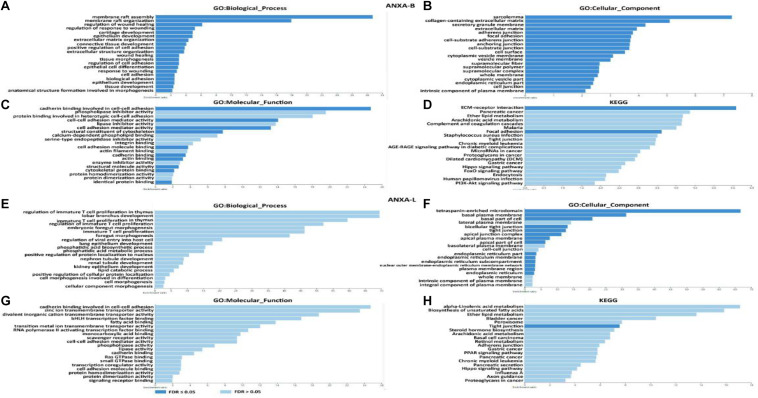
Function enrichment of ANXA family members in BC. **(A–D)** GO and KEGG pathway analysis in ANXA-B. **(E–H)** GO and KEGG pathway analysis in ANXA-L.

Based on the above results, ANXA1, ANXA2, ANXA3, ANXA5, ANXA6, ANXA8, and ANXA13 were identified as potential genes related to basal-subtype bladder cancer (ANXA-B), and ANXA4, ANXA9, ANXA10, and ANXA11 were defined as luminal-subtype bladder cancer-related genes (ANXA-L). On this basis, we further explored the potential mechanisms of ANXA-B and ANXA-L in bladder cancer. The 25 most significantly positively correlated genes of each ANXA member were analyzed using the cBioPortal platform. WebGestalt was then used to perform GO and KEGG analyses of 200 co-expressed genes of ANXA-B and 100 co-expressed genes of ANXA-L to explore upregulated pathways of the ANXA proteins in the two subtypes of bladder cancer (Figure 8 and Supplementary Table 2). In the GO analysis, we selected 20 of the most significant biological process, cellular component, and molecular function GO terms. Regarding BP terms, there was significant enrichment of adhesion regulation genes, such as the genes in GO:0045785, GO: 0030155 (regulation of cell adhesion), GO: 0007155 (cell adhesion), and GO: 0022610 (biological adhesion). Processes related to injury healing (GO:0061041, regulation of response to wounding; GO:0042060, wound healing; and GO:0009611, response to wounding) and membrane raft assembly (GO:0001765, membrane raft assembly; and GO:0031579, membrane raft organization) were also significantly regulated by ANXA-B. Regarding CC terms, ANXA-B related co-expressed genes were mainly found to play a role in cell matrix and cell connection functions: GO:0062023 (collagen-containing extracellular matrix), GO:0031012 (extracellular matrix), GO:0005912 (adherens junction), GO:0005925 (focal adhesion), GO:0005924 (cell-substrate adherens junction), GO:0070161 (anchoring junction), GO:0030055 (cell-substrate junction), GO:0009986 (cell surface), and GO:0030054 (cell junction). Regarding MF terms, the dark blue bar represents FDR ≤ 0.05. Related functions mainly involved cell adhesion and cytoskeleton structure: GO:0098641 (cadherin binding involved in cell-cell adhesion), GO:0098632 (cell-cell adhesion mediator activity), GO:0098631 (cell adhesion mediator activity), GO:0005200 (structural constituent of cytoskeleton), GO:0050839 (cell adhesion molecule binding), GO:0045296 (cadherin binding), GO:0003779 (actin binding), GO:0005198 (structural molecule activity), GO:0008092 (cytoskeletal protein binding), and GO:0042802 (identical protein binding). In the KEGG analysis, ANXA-B-related genes were mainly expressed in the HSA04512 (ECM-receptor interaction) and HSA04510 (focal adhesion) pathways, and the P and FDR values were both <0.05. We also analyzed the possible role of ANXA-L (ANXA4, ANXA9, ANXA10, and ANXA11) in the regulation of luminal bladder cancer. Regarding BP terms, the immune regulation of T cells was enriched (GO:0033084, regulation of immature T cell effects in thymus; GO:0033080, immature T cell effects in thymus; GO:0033083, regulation of immature T cell effects; GO:0033079, immature T cell effects and cellular morphogenesis involved in differentiation; GO:0000904, cell morphogenesis; GO:0032989, cellular morphogenesis and component morphogenesis). Regarding CC terms, related genes were mainly involved in the maintenance of plasma membrane structure and function: GO:0009925 (basal plasma membrane), GO:0016328 (lateral plasma membrane), GO:0016324 (apical plasma membrane), GO:0016323 (basolateral plasma membrane), GO:0098590 (activity and the components of plasma membrane), GO:0005887 (integral component of plasma membrane), GO:0044432 (endoplasmic reticulum part), GO:0005789 (endoplasmic reticulum membrane), and GO:0098827 (endoplasmic reticulum subcompartment). Regarding MF terms, ANXA-L were found to perform binding functions and signal transduction: binding to transcription factors (GO:0043425, bHLH transcription factor binding); binding to fatty acids (GO:0005504, fatty acid binding); binding to monocarboxylic acid (GO:0033293, monocarboxylic acid binding); binding to cadherin (GO:0045296, cadherin binding); binding to Ras GTPase (GO:0017016, Ras GTPase binding); binding to small GTPase (GO:0031267, small GTPase binding); binding to cell adhesion molecules (GO:009864, cadherin binding involved in cell-cell adhesion); binding to transcription factors (GO:0050839, cell adhesion molecule binding); and binding to signal receptors (GO:0005102, signaling receptor binding). Ultimately, only the HSA04530 pathway (involving tight junctions) P and FDR values were both <0.05 in the KEGG analysis.

### Correlation Analysis Between ANXA Members and Immune Cell Infiltration

**FIGURE 9:**
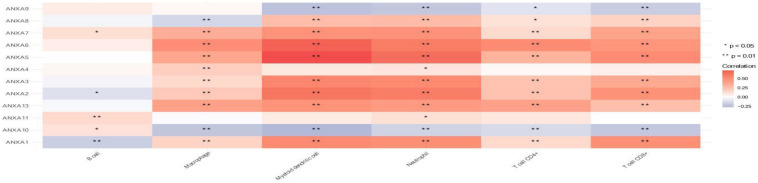
Association of mRNA expression of ANXA family members with immune infiltration level in BC. (*p < 0.05; **p < 0.01).

Finally, we analyzed the relationship between ANXA family member expression and the infiltration of immune cells in bladder cancer (Figure 9). The TIMER2.0 platform can be used to perform an analysis of the correlation between gene expression and the degree of infiltration of six major immune cells in the tumor microenvironment. ANXA1–3 and ANXA5–7 were significantly positively correlated with the infiltration of five kinds of immune cells (macrophages, myeloid dendritic cells, neutrophils, CD4+ T cells, and CD8+ T cells). ANXA8 had similar characteristics except that it was negatively correlated with macrophages. On the other hand, ANXA9 and ANXA10 showed opposite results and significantly negative correlations with 4 types of immune cells (myeloid dendritic cells, neutrophils, CD4+ T cells, and CD8+ T cells).

## Discussion

Since bladder cancer is the most common disease of the urinary system, understanding the biological mechanism of bladder cancer is crucial for urological research. In this article, we first studied the expression of different ANXA family members in bladder tumor and normal tissues, and the results showed that the mRNA of ANXA2, ANXA3, ANXA4, ANXA8, and ANXA9 were significantly increased in tumors, while the mRNA of ANXA6, ANXA7, and ANXA11 were significantly decreased in tumor tissues. Furthermore, we analyzed the expression of annexin proteins in NMIBC and MIBC. As shown in Supplementary Table 1, ANXA1, ANXA2, ANXA3, ANXA5, ANXA6, ANXA7, and ANXA8 were mainly highly expressed in MIBC, while ANXA9, ANXA10, and ANXA11 were significantly higher in the NMIBC group. Considering that MIBC and NMIBC have considerable differences in biological traits, these results suggest that ANXA proteins may be involved in phenotypic maintenance and functional regulation in different types of bladder cancer. Annexins are calcium-binding proteins with a phospholipid-binding domain that play an important role in the regulation of intercellular inflammation, cell signaling, and cell proliferation and differentiation (Smith and Moss, 1994; Han et al., 2020). Previous studies have shown that ANXA family proteins can regulate cancer and can be used as prognostic markers (Patton et al., 2005; Lecona et al., 2008; Biaoxue et al., 2012; Moghanibashi et al., 2012; Suo et al., 2012; Yu et al., 2014; Yamanoi et al., 2019). In our study, after analyzing the expression of ANXA proteins in bladder cancer, we further explored their expression levels in different molecular subtypes of MIBC for the first time. Most importantly, we found that annexins were closely related to tumor phenotype and histological morphology in bladder cancer. Compared with that in papillary bladder tumors, the protein expression of ANXA1, ANXA2, ANXA3, ANXA5, ANXA6, ANXA7, ANXA8, and ANXA13 was especially increased in non-papillary bladder tumors. In addition, ANXA9, ANXA10, and ANXA11 were highly expressed in papillary bladder cancer, and ANXA10 showed the most significant differences. These results suggest that different annexins may play a crucial role in regulating the differentiation of tumors and have potential value as biomarkers and therapeutic targets. In Bizzarro’s study (Belvedere et al., 2014), knockdown of the expression of ANXA1 inhibited the differentiation of myoblasts to myocytes. In Wang’s study, ANXA1 was found to be highly expressed in normal cervical squamous tissue, and its expression was significantly reduced as the tissue morphology changed with the progression of cervical tumors (Wang et al., 2008). Another study by Liu reported that only ANXA10 of the ANXA family proteins was highly expressed in papillary thyroid carcinoma and was related to papillary thyroid carcinoma differentiation and progression (Liu et al., 2021). Interestingly, we found that bladder cancer with high expression of ANXA1 also showed a squamous pattern, while bladder cancer with high expression of ANXA10 showed a papillary pattern.

Non-muscle-invasive bladder cancer generally shows papillary characteristics, while the histological phenotypes of MIBC are more diverse. The University of North Carolina (UNC), MD Anderson Cancer Center, and TCGA have all conducted their own studies on MIBC subtype identification. The UNC team proposed that bladder cancer is similar to breast cancer and used unsupervised consensus clustering to divide samples into “basal” and “luminal” categories. Similarly, in the studies of MD Anderson Cancer Center and TCGA in 2014, MIBC was mainly divided into these two categories (Choi et al., 2014; Damrauer et al., 2014). Based on research performed in 2014, TCGA updated the molecular subtypes of MIBC in 2017, and the luminal subtype was subdivided into luminal-papillary, luminal, and luminal-infiltrated subtypes, and the basal subtype was divided into basal-squamous and neuronal subtypes. The general characteristics of the luminal subtype shows positivity for FOXA1, GATA3, and KRT20, while the basal subtype lacks FOXA1 and GATA3 expression and is positive for KRT5, KRT6, and KRT14 (Robertson et al., 2017). In our study, TCGA was used to analyze the correlation between ANXA family proteins and characteristic luminal or basal subtype genes. The results showed that the expression of ANXA1, ANXA2, ANXA3, ANXA5, ANXA6, ANXA7, ANXA8, and ANXA13 was negatively correlated with the expression of FOXA1, GATA3, and KRT20 and positively correlated with the expression of KRT5, KRT6, and KRT11, while the expression of ANXA4, ANXA9, ANXA10, and ANXA11 showed the opposite patterns. The results suggest that ANXA-B (ANXA1, ANXA2, ANXA3, ANXA5, ANXA6, ANXA7, ANXA8, and ANXA13) and ANXA-L (ANXA4, ANXA9, ANXA10, and ANXA11) may be involved in the functional regulation of these characteristic genes. In Wang’s study, ANXA2 was found to directly bind to STAT3, enhance its transcriptional activity and promote EGF-induced EMT (Wang et al., 2015). Similarly, EGF receptors and STAT3 are overexpressed in basal-squamous bladder tumors, accompanied by high expression of ANXA2. Whether ANXA2 plays the same role in bladder tumors needs further study.

In bladder cancer, NMIBC includes the Ta, T1, and CIS stages, while T2–T4 bladder cancer is characterized by invasion into the muscle layer, also known as MIBC. In this study, we also analyzed the correlation between ANXA expression and the clinical stage of bladder cancer. The results showed that the expression levels of ANXA2, ANXA5, and ANXA6 were significantly positively correlated with clinical stage. In the analysis of overall survival, patients with high expression of ANXA1, ANXA2, ANXA3, ANXA5, ANXA6, and ANXA7 had poor prognosis, while those with high expression of ANXA9 had longer overall survival. In view of the above results, ANXA2, ANXA5, and ANXA6 have utility for the categorization of pathological stage and prediction of overall survival in bladder cancer and can reflect the progression and prognosis of bladder cancer. In Yuan’s study on gastric cancer, the survival rate of patients with positive expression of annexin A7 was lower than that of patients with negative expression (Yuan et al., 2019). In addition, ANXA1 is able to promote cell migration, invasion, and angiogenesis and participate in pancreatic cancer progression (Pessolano et al., 2018). The same ANXA may have the opposite prognostic effect in different cancers. Patients with colorectal cancer with high ANXA9 expression had statistically relatively worse prognosis (Miyoshi et al., 2014). However, in our study, patients with higher ANXA9 expression were more likely to show luminal-subtype bladder cancer and have a better prognosis.

In further analysis of the function of ANXA genes, we found that the functions of ANXA-B (ANXA1, ANXA2, ANXA3, ANXA5, ANXA6, ANXA7, ANXA8, and ANXA13) co-expressed genes were mainly related to cell adhesion, damage repair, cytoskeleton composition and extracellular matrix interactions. On the other hand, the enriched functions of ANXA-L (ANXA4, ANXA9, ANXA10, and ANXA11) were mainly related to the regulation of immature T cells, cellular morphogenesis, binding transduction, plasma membrane function and tight junctions. Cytoskeletal-associated proteins play an active role in coordinating the adhesion and migration machinery in cancer progression, and targeting annexin A2 could effectively inhibit tumor cell adhesion, migration and in vivo grafting (Staquicini et al., 2017). In addition, studies have demonstrated the function of ANXA2 in regulating the adhesion of hematopoietic stem cells to osteoblasts and bone marrow endothelial cells and the adhesion of prostate cancer cells to endothelial cells (Jung et al., 2007; Shiozawa et al., 2008). ANXA repair functions also play an important role in tumor renewal and growth. In breast cancer, studies have proven that ANXA2, ANXA4, ANXA5, ANXA6, and ANXA7 are required for repair in breast cancer cells, indicating that a network of annexins participate in the plasma membrane repair response (Sønder et al., 2019). In Hannah L’s study, ANXA1 was able to protect against DNA damage and promote modulation of cell adhesion and motility, indicating its potential role in cancer initiation and progression in breast carcinoma (Swa et al., 2012). ANXA proteins also determine the morphogenesis of cells. Through an adherens junction-mediated pathway upstream of Akt, endothelial morphogenesis could be regulated by ANXA2 (Su et al., 2010). Mechanistic studies of human mammary cells and mammary glands of mice showed that ANXA8 upregulation is caused by genetic mutations affecting RARA functions and affects postnatal developmental processes such as myelopoiesis and mammary gland morphogenesis (Rossetti and Sacchi, 2020). Annexins also exhibit anti-inflammatory and proinflammatory effects in a variety of inflammatory experimental models. For example, ANXA1 can regulate the ERK/MAPK signaling pathway, which affects the activity, proliferation, and differentiation of T cells and exerts corresponding anti-inflammatory effects. ANXA1 can also be phosphorylated by PKC, resulting in the induction of proinflammatory cytokines (Shao et al., 2019). In Qiu’s study, an elevated ANXA2 level resulted in the upregulation of the proportion of Treg cells and promoted tumor immune escape (Qiu et al., 2020). Annexin A1 is a key functional player in tumor development, linking the various components in the tumor stroma by its actions in endothelial cells and immune cells (Yi, 2012). In our analysis of the relationship between ANXA expression and immune cell infiltration, we found high infiltration of myeloid dendritic cells, neutrophils, CD4+ T cells and CD8+ T cells when ANXA-B (1, 2, 3, 5, 6, 7, 8, and 13) was overexpressed. The expression levels of ANXA9 and ANXA10 were significantly negatively correlated with the degree of infiltration of the above immune cells, suggesting that patients with BC with high expression of ANXA-B may be more suitable for immunotherapy.

## Conclusion

In this study, we analyzed the expression characteristics and prognostic utility of ANXA family proteins in bladder cancer for the first time. In addition, we also determined the expression characteristics of ANXA family members in different subtypes of MIBC. The results suggest that differences in the expression of ANXA family members are closely related to the histological morphology of bladder cancer. It should be noted that ANXA1, ANXA2, ANXA3, ANXA5, ANXA6, ANXA7, ANXA8, and ANXA13 are mainly expressed in basal-subtype MIBC, while ANXA4, ANXA9, ANXA10, and ANXA11 are mainly expressed in luminal-subtype MIBC. These findings provide more markers for subtype identification and are conducive to further mechanistic research. It’s important to point out that our data only indicate the correlation between ANXA family proteins and BC. Further experiments are needed to verify the protein expression level in tissues and its functional role in BC. In summary, ANXA family proteins can serve as potential markers for the identification and prognostication of bladder cancer.

## Data Availability Statement

The datasets presented in this study can be found in online repositories. The names of the repository/repositories and accession number(s) can be found in the article/Supplementary Material.

## Author Contributions

WW and GJ developed the idea, data analysis, and writing original draft. LC contributed to data curation and formal analysis. HL and SX reviewed the manuscript. All authors read and approved the final manuscript.

## Conflict of Interest

The authors declare that the research was conducted in the absence of any commercial or financial relationships that could be construed as a potential conflict of interest.

## Publisher’s Note

All claims expressed in this article are solely those of the authors and do not necessarily represent those of their affiliated organizations, or those of the publisher, the editors and the reviewers. Any product that may be evaluated in this article, or claim that may be made by its manufacturer, is not guaranteed or endorsed by the publisher.

## Footnotes

1
http://ualcan.path.uab.edu/

2
http://www.oncomine.org

3
http://www.proteinatlas.org

4
http://kmplot.com/analysis/

5
http://timer.comp-genomics.org/

6
http://www.cbioportal.org

7
http://www.webgestalt.org
